# Quality Matters? The Involvement of Mitochondrial Quality Control in Cardiovascular Disease

**DOI:** 10.3389/fcell.2021.636295

**Published:** 2021-03-22

**Authors:** Kai-Lieh Lin, Shang-Der Chen, Kai-Jung Lin, Chia-Wei Liou, Yao-Chung Chuang, Pei-Wen Wang, Jiin-Haur Chuang, Tsu-Kung Lin

**Affiliations:** ^1^Center for Mitochondrial Research and Medicine, Kaohsiung Chang Gung Memorial Hospital and Chang Gung University College of Medicine, Kaohsiung, Taiwan; ^2^Department of Anesthesiology, Kaohsiung Chang Gung Memorial Hospital and Chang Gung University College of Medicine, Kaohsiung, Taiwan; ^3^Department of Neurology, Kaohsiung Chang Gung Memorial Hospital and Chang Gung University College of Medicine, Kaohsiung, Taiwan; ^4^Center of Parkinson’s Disease, Kaohsiung Chang Gung Memorial Hospital and Chang Gung University College of Medicine, Kaohsiung, Taiwan; ^5^Department of Metabolism, Kaohsiung Chang Gung Memorial Hospital and Chang Gung University College of Medicine, Kaohsiung, Taiwan; ^6^Department of Pediatric Surgery, Kaohsiung Chang Gung Memorial Hospital and Chang Gung University College of Medicine, Kaohsiung, Taiwan

**Keywords:** mitochondria, mitophagy, cardiovascular disease, nucleus, hypertension, ischemic heart, diabetic cardiomyopathy, mitochondrial haplogroup

## Abstract

Cardiovascular diseases are one of the leading causes of death and global health problems worldwide. Multiple factors are known to affect the cardiovascular system from lifestyles, genes, underlying comorbidities, and age. Requiring high workload, metabolism of the heart is largely dependent on continuous power supply via mitochondria through effective oxidative respiration. Mitochondria not only serve as cellular power plants, but are also involved in many critical cellular processes, including the generation of intracellular reactive oxygen species (ROS) and regulating cellular survival. To cope with environmental stress, mitochondrial function has been suggested to be essential during bioenergetics adaptation resulting in cardiac pathological remodeling. Thus, mitochondrial dysfunction has been advocated in various aspects of cardiovascular pathology including the response to ischemia/reperfusion (I/R) injury, hypertension (HTN), and cardiovascular complications related to type 2 diabetes mellitus (DM). Therefore, mitochondrial homeostasis through mitochondrial dynamics and quality control is pivotal in the maintenance of cardiac health. Impairment of the segregation of damaged components and degradation of unhealthy mitochondria through autophagic mechanisms may play a crucial role in the pathogenesis of various cardiac disorders. This article provides in-depth understanding of the current literature regarding mitochondrial remodeling and dynamics in cardiovascular diseases.

## Introduction

**FIGURE 1:**
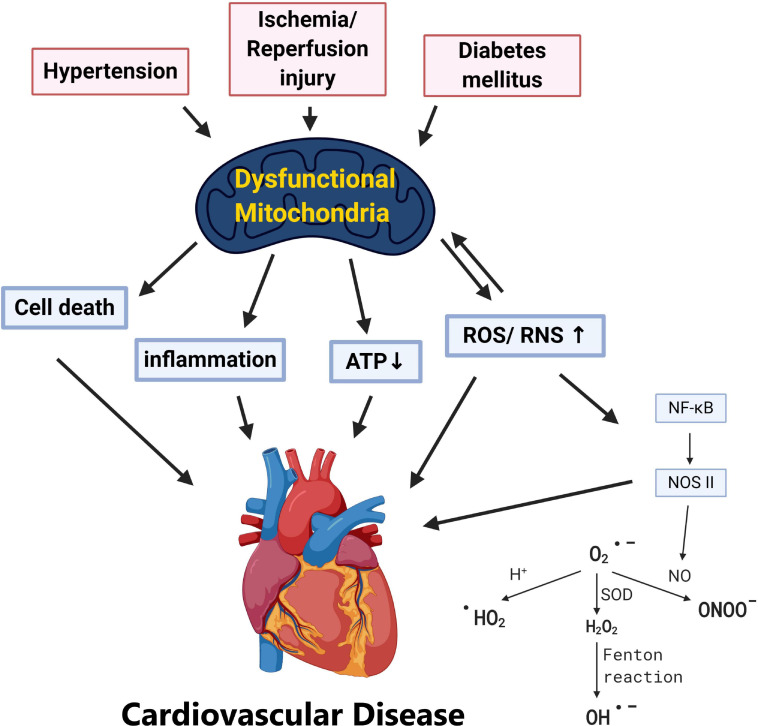
Pathological stress including hypertension, ischemia/reperfusion, and diabetes mellitus results in dysfunctional mitochondria. Various cardiovascular risk factors including hypertension, ischemia/reperfusion injury, and diabetes leads to mitochondrial dysfunction. Without adequate quality control of the damaged mitochondria, this may result in (1) ATP depletion, (2) overproduction of ROS/RNS, (3) mitochondria-dependent cell death (apoptosis), and (4) systemic inflammation which provokes cardiovascular pathogenesis.

Cardiovascular diseases are one of the leading causes of death worldwide causing global health problems (Yusuf et al., 2001). Multiple factors are known to affect the cardiovascular system from lifestyles, genes, epidemiological transition, and age (Gluckman et al., 2009). Biological aging has long been known as an inevitable risk factor of cardiovascular diseases, and it has been suggested that pathogenesis of cardiovascular dysfunction may be related to inflammation, oxidative stress, DNA damage, telomere shortening, lipotoxicity, and mitochondrial damage (Gluckman et al., 2009; Kubben and Misteli, 2017; Wu et al., 2019). With the overwhelming workload and energy required, metabolism of the heart is largely dependent on mitochondria, the major and most efficient producers of cellular adenosine triphosphate (ATP) (Wu et al., 2019). In addition to being major cellular power plants through oxidative phosphorylation (OXPHOS), mitochondria also serve as sites for β-oxidation, the Krebs cycle, calcium reservoirs, the initiators of the intrinsic apoptotic pathway, and the regulators of necrosis (Spinelli and Haigis, 2018). Thus, these highly dynamic and complex organelles have gained huge attention among researchers and accumulating data have advocated the critical roles of mitochondrial function in various aspects of cardiovascular pathology including: hypertension (HTN), atherosclerosis, ischemia/reperfusion (IR) injury, and type 2 diabetes mellitus (T2DM) (Fearon and Faux, 2009; Ong and Hausenloy, 2010; Vasquez-Trincado et al., 2016; Figure 1).

Mitochondria are essential for the functioning of cardiomyocytes where they occupy around 30% of the total cell volume and generate up to 30 kg of ATP per day (Lin K.J. et al., 2019). A double membrane organelle, the mitochondrion is composed of a mitochondrial outer membrane (MOM), an intermembrane space (IMS), and the convoluted mitochondrial inner membrane (MIM) surrounding the central mitochondrial matrix. It is on the MIM that the OXPHOS pathway takes place and where most of the ATP production occurs, while the tricarcoxylic acid (TCA) cycle in the mitochondrial matrix supports the fuel for this machinery (Hall et al., 2014). The OXPHOS pathway produces more than 95% of cellular energy via the electron transport chain (ETC) and involves five protein complexes assembled on the MIM. During the OXPHOS process, membrane potential is generated across the MIM. Abnormal membrane potential may signal the cell to undergo various stress responses and even mitochondrial-mediated apoptosis. Reactive oxygen species (ROS) are generated during OXPHOS when electrons leak out during electron transportation from the ETC to oxygen. Generated as metabolic byproducts, ROS are highly reactive chemical molecules formed due to the electron acceptability of O_2_ and perform a dual role as harmful, protective, or signaling factors according to the balance of ROS production and disposal (Droge, 2002). Highly reactive with unpaired electrons over their molecular outer layer, at unbalanced conditions, these ROS can cause DNA and RNA damage, lipid peroxidation, protein carbonylation, imbalance cellular redox and irreversible impairment of the mitochondria, and eventually cellular death and cardiovascular pathology (Zhao et al., 2019).

Since mitochondrial function is crucial for normal cellular activity, strict mitochondrial quality control through coordination of various processes such as proteostasis, morphology regulation, and autophagy to ensure cellular homeostasis is key to exploiting the pathogenesis of cardiovascular diseases (Hammerling and Gustafsson, 2014; Anzell et al., 2018; Picca et al., 2018; Tahrir et al., 2019). A dynamic organelle, the mitochondrion forms networks to maintain its integrity and interchange mitochondrial material in response to cellular stress through the process of fusion. On the other hand, damaged mitochondria can be dispatched through fission where damaged parts could be degraded. Hence, mitochondria undergo continuous dynamic and morphological alterations in response to a stressed cellular environment (Eisner et al., 2018). Dysfunctional mitochondria can then be efficiently degraded through the process of mitochondrial autophagy, mitophagy.

As healthy mitochondria may be essential for maintaining normal cardiovascular function, further treatments targeting mitochondrial quality control may act as a preventive strategy for these notorious clinical conditions. Thus in this review, we will focus on mitochondrial quality control concerning the pathophysiology of cardiovascular diseases.

## Mitochondrial Biology

Mitochondria have long been the subject of intense investigation due to their multiple roles in the cellular survival of eukaryocytes (Duchen, 2004; Van Remmen and Jones, 2009; Wang and Youle, 2016). In the following section, we will describe this organelle in different aspects including mitochondrial genetics, the OXPHOS system, and mitochondria-related programmed cell death.

### Mitochondrial Genetics

According to endosymbiotic theories, mitochondria were once parasitic bacteria, later becoming the semi isolated entities in the cell. Like their prokaryote ancestors, mitochondria contain their own hereditary material, the double-stranded and circular mitochondrial DNA (mtDNA). Highly compact with only two regulatory regions and exons without introns, mtDNA contains 37 genes encoding 22 transfer RNAs, two ribosomal RNAs, and 13 polypeptides that are crucial subunits belonging to the enzyme complexes of the OXPHOS system (Wiedemann and Pfanner, 2017). Following a maternal pattern of inheritance, the mitochondria genome resides outside of the nucleus and relies immensely on products encoded in the nuclear DNA (Taylor and Turnbull, 2005; Jang and Lim, 2018). Mitochondria are major sites of ROS production under physiological conditions, and the structure of the mtDNA molecule is similar to bacteria with only limited self-repairing systems; therefore, mtDNA are prone to mutation generation under consistent attacks of free radicals produced *in situ* compared to the nuclear genome (Chinnery et al., 1999). With around 2–10 copies of mtDNA present in each mitochondrion and upward of 100s and 1,000s of mitochondria in each cell, a heterogenous mixture of mutations in mtDNA-encoded genes with wild-types can be present, known as heteroplasmy (Stewart and Chinnery, 2015). The vast majority of these mitochondrial genetic alterations are not directly tied to pathogenesis of diseases. Through human evolution, some mtDNA variants are preferentially passed down the maternal line (Stewart and Chinnery, 2021) and different combinations of single nuclear polymorphisms (SNPs) in mtDNA inherited from a common ancestor can be defined into mitochondrial haplogroups, which can be used to represent genetic populations on the mitochondrial phylogenetic tree (Kazuno et al., 2006). mtDNA variants have attracted special attention due to their potential for affecting redox homeostasis and causing alteration of mitochondrial function. Mitochondrial haplogroups, considered to represent genetic populations on the mitochondrial phylogenetic tree can also be utilized as biomarkers for disease association. Recently, Liou et al. (2016) reported that the mitochondrial haplogroup B5 is resistant to the development of PD in Taiwanese people of ethnic Chinese background (Liou et al., 2016). In their cellular studies, the B5 cybrid, containing G8584A/A10398G variants, showed more resistance to the complex I inhibitor, rotenone, with lower ROS generation and apoptosis rate than the B4 cybrid which does not harbor these variants (Liou et al., 2016). These results further provide direct genetic and functional evidence that mtDNA variations and consequential mitochondrial function alternation can potentially influence the risks of clinical diseases. Fetterman et al. (2013) also demonstrated in a mitochondrial-nuclear exchange mouse model of mtDNA polymorphisms that mtDNA genetic background significantly modulated mitochondrial bioenergetics, cellular ROS production, and susceptibility to cardiac volume overload, independent of nuclear background.

### Mitochondrial Oxidative Phosphorylation and ROS Production

A structurally two membraned organelle, the mitochondrion harbors two compartments, the central matrix and the IMS with the MIM in between and the MOM as the outermost layer. The highly folded MIM forms the cristae housing the OXPHOS machinery, which is comprised of the electron transport chain (ETC), complex I (CI) to complex IV (CIV), and F1F0-ATP synthase (complex V). Using NADH as a substrate for CI and succinic acid for CII, the ETC complexes transfer electrons from electron donors to electron acceptors via redox reactions which generates energy and drives protons from the matrix side of CI, CIII, and CIV across the MIM into the IMS side. Therefore, an electro-chemical gradient is generated across the MIM with accumulation of protons pumped into the IMS and generates mitochondrial membrane potential (ΔΨ). This electro-chemical gradient driven by substrate oxidation is then coupled to the synthesis of ATP with phosphorylation of ADP at complex V, the process is called OXPHOS (Schumacker et al., 2014; Zhao et al., 2019).

In most circumstances, the oxygen molecule acts as the final acceptor of electrons at complex IV to form harmless water. However, both as a consequence of normal electron transport or during mitochondrial dysfunction, electrons can leak from the ETC, especially at complexes I and III, to oxygen to generate superoxide anions (O_2_^•–^) (Gorrini et al., 2013; Liemburg-Apers et al., 2015). O_2_^•–^ can then be converted into hydrogen peroxide (H_2_O_2_) by superoxide dismutase (SOD), and later to hydroxyl radicals (HO) via the Fenton reaction (Thomas et al., 2009; Zorov et al., 2014). Interaction of O_2_^•–^ with protons can also generate hydroperoxyl radicals (HOO). ROS are highly reactive and cause oxidative damage to macromolecules (Lin et al., 2018, Lin K.J. et al., 2019). Since unbalanced high ROS concentrations are toxic, mitochondria possess powerful antioxidant systems to prevent excess oxidative stress induction (Zorov et al., 2014). Mitochondrial antioxidant defenses include antioxidative enzymes such as glutathione reductase, glutathione peroxidase, SOD (the Mn-dependent isoform (Mn SOD, SOD2) in the mitochondrial matrix and the Cu,Zn-dependent isoform (Cu,Zn SOD, SOD1) in the IMS and cytosol), thioredoxin, peroxiredoxins, and catalase (Mailloux, 2018). Under physiological conditions, the balance between ROS generation and ROS scavenging is highly regulated to provide efficient detoxification. However, oxidative stress can occur when ROS production overwhelms these defense systems leading to organelle damage (Richter, 1995). Therefore, mitochondria are more susceptible to the accumulation of damaged mtDNA and vulnerable to oxidative damage, as they are not only constantly surrounded and attacked by the ROS produced in situ, but also have an insufficient DNA repair system due to limited organelle genomic capacity (Druzhyna et al., 2008).

### Mitochondria-Dependent Apoptosis

Mitochondria also play a central role in the regulation of apoptotic cell death. Apoptosis can be initiated through extrinsic or intrinsic mitochondrial pathways (Lopez and Tait, 2015). Under excessive cellular stress, activation of intrinsic pathways causes mitochondrial outer membrane permeabilization (MOMP) which then causes the release of the apoptotic signal cytochrome *c* from the intermembrane space into the cytosol. Once released, cytochrome *c* binds to apoptotic protease activating factor-1 (APAF -1) forming the structure apoptosome. Apoptosome in turn recruits and activates pro-caspase-9, which then cleaves and activates caspase-3 and caspase-7, the major executors of apoptosis. The activation of caspase proteases subsequently cleaves up to hundreds of proteins of the cell causing programmed cell death (Taylor et al., 2008; Tait and Green, 2010). Other pro-apoptotic proteins released from the mitochondrial IMS into the cytoplasm includes the second mitochondria-derived activator of caspases (SMAC) and Omi which antagonize endogenous inhibitors of caspase function called the X-linked inhibitor of apoptosis protein (XIAP) (Suzuki et al., 2004; Hui et al., 2011; Vasudevan and Ryoo, 2015; Singh et al., 2019; Tang et al., 2019). In the intrinsic mitochondrial apoptosis pathway, the B-cell lymphoma 2 (BCL-2) family consists of both apoptotic and anti-apoptotic proteins and regulates MOMP (Kalkavan and Green, 2018; Singh et al., 2019). BCL-2 effector proteins modulate the activation of the pro-apoptotic process including BCL-2-associated X protein (BAX), BCL-2 antagonist or killer (BAK), and BH3 interacting domain death agonist (Bid) and antiapoptotic signals including BCL-2, B-cell lymphoma-extra large (BCL-xL), and myeloid cell leukemia 1 (MCL-1) (Kalkavan and Green, 2018; Malina et al., 2018). The MOMP executioner, BAX is translocated to the MOM and BAK must be disengaged from antiapoptotic BCL-2 proteins (MCL-1 and BCL-xL) to activate cellular apoptosis. After activation of either or both BAX or BAK results in the proteins forming pores on the MOM and cause MOMP (Singh et al., 2019). These BCL-2 family proteins also take part in the extrinsic pathway which is typically activated through the binding of a subset of the TNF receptor superfamily such as tumor necrosis factor 1 (TNFR1), TNF-related apoptosis-inducing ligand (TRAIL) receptors (DR4 and DR5), and Fas (Apo-1; CD95) (Ping et al., 2015). A cross-talk between extrinsic and intrinsic pathways is needed to ensure the process of the extrinsic death pathway. With the binding of death ligands on these receptors, caspase-8 is activated and then further engagement of caspase-3 and caspase-7 occurs. Yet in some cells such as hepatocytes and pancreatic cells, pathways lacking MOMP is not enough to bring about cell death. In such occasions caspase-8 will cleave Bid, a BCL-2 family protein, to truncated Bid (tBid), which in turn activates BAX and BAK proteins to activate MOMP and eventually cell death (Wang and Youle, 2009; Singh et al., 2019). Under extreme environments, damaged mitochondria can behave like cellular sensors to activate intrinsic death signals leading to the point of no return with mitochondrial quality control being crucial for cellular survival.

## Mitochondrial Quality Control

**FIGURE 2:**
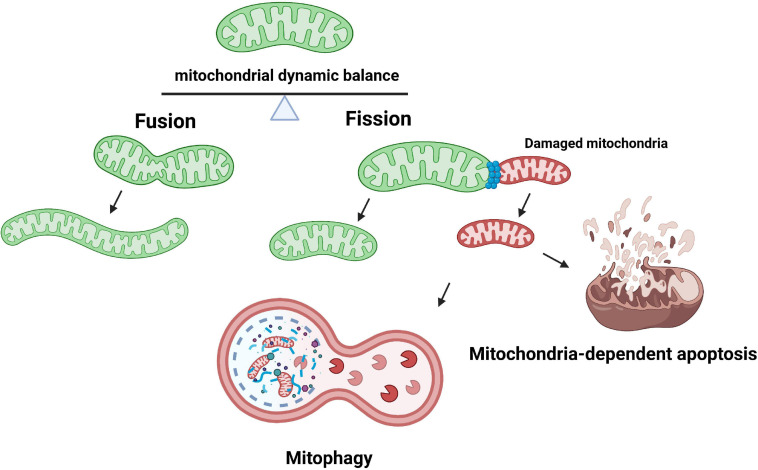
Mitochondrial dynamics and quality control. Mitochondria fuse to merge intra-organelle contents including the mitochondrial DNA and prevent permanent loss of essential components. The fusion process is mediated by the GTPase proteins mitofusins 1 and 2 (MFN1/2) on the mitochondrial outer membrane, and optic atrophy type 1 (OPA1) on the mitochondrial inner membrane. Mitochondrial fission, on the other hand, can create new mitochondria or enable quality control by segregating damaged mitochondria for subsequent degradation via mitophagy. The fission process is primarily carried out by dynamin-related protein 1 (DRP1) and mitochondrial outer membrane mitochondrial fission 1 protein (FIS1). If mitochondria quality control is dysfunctional, the mitochondria may induce mitochondria-dependent apoptosis.

Dysfunction of mitochondria caused by oxidative stress has severe cellular consequences linked to human diseases. Several surveillance strategies have evolved to limit mitochondrial damage and ensure cellular survival. As previously mentioned, controlling the abundances of ROS production is the first line of defense. However, when damage has occurred, a secondary set of repair pathways take place to ensure the normal functionality of molecules. For example, the methionine sulfide reductase (Msr) system that consists of MsrA and MsrB reduces a subset of oxidized proteins, namely oxidized methionine moieties, back to their reduced methionine. Also, chaperones contribute through refolding the damaged misfolded proteins back to their native three dimensional structures (Fischer et al., 2012; Penna et al., 2018). In spite of all these protective and repair mechanisms, the vast majority of damaged proteins cannot be efficiently repaired. The accumulation of irreversibly damaged components fosters further acceleration of mitochondrial dysfunction and subsequent cell death; therefore, the removal of these denatured proteins is essential. Proteolysis and removal of these dysfunctional proteins through the cytosolic ubiquitin/26S proteasome system (UPS) has been shown to be a crucial part in the quality control of mitochondrial proteins (Tatsuta and Langer, 2008). Apart from cytosolic UPS, mitochondria quality is largely regulated by mitochondrial dynamics and mitophagy. The processes of mitochondrial fusion facilitate the redistribution of mitochondrial components while fission (division) allows for damaged mitochondria to be degraded through fragmentation. The process of mitophagy is responsible for the degradation and recycling of damaged mitochondria (Figure 2). Recently, these mitochondrial quality control mechanisms have been shown to be heavily involved in various cardiovascular pathological conditions (Anzell et al., 2018).

### Mitochondrial Fusion and Fission

Mitochondria form highly dynamic and intricate networks, balancing on the opposing processes of morphological changes: mitochondrial fission and fusion (Parra et al., 2011; Lin K.J. et al., 2019). The mitochondria fusion process is primary regulated by three dynamin-related guanosine triphosphatases (GTPases) called mitofusin 1, mitofusin 2 (MFN1 and MFN2), and optic atrophy protein 1 (OPA1). In addition to the regulation of mitochondrial fusion, outer membrane MFN1 and MFN2 have been revealed to undertake ER-associated degradation (ERAD) through polyubiquitination by the E3 ubiquitin ligase parkin and thus stimulate damaged mitochondrial protein degradation through UPS (Picca et al., 2018). On the other hand, mitochondrial fission 1 protein (FIS1) and dynamin-related protein 1 (DRP1) regulates mitochondrial fission (Ishihara et al., 2004; Lin et al., 2018). Mitochondria fusion describes the fusion of two neighboring mitochondria to allow the sharing of metabolites, enzymes, and genetic materials. This process involves the integration of MFN1 with MFN 2 (heterodimers) or MFN2 with MFN2 (homodimers) to achieve fusion of the MOM (Ishihara et al., 2004; Chen and Chan, 2009; Westermann, 2012; Hall et al., 2014), while Opa1 mediates the fusion of the MIM as well as maintaining the normal inner membrane cristae structure (Malka et al., 2005; Song et al., 2007). Such an act preserves mitochondria membrane integrity and membrane permeability, improving mitochondrial resistance to injury from oxidative damage. Meanwhile, damaged mitochondria can be removed through mitochondrial fission, the division or fragmentation from one mitochondria to two (Chen and Chan, 2009; Ong et al., 2010; Elgass et al., 2013; Otera et al., 2013). This process is regulated by GTPase DRP1, mitochondrial FIS1, and mitochondrial fission factor (MFF). FIS1 acts as an inhibition factor of mitochondrial fusion and also as a receptor for the recruitment of fission-related proteins such as DRP1 to the outer mitochondrial membrane, where it initiates cleavage of the mitochondria by interaction with FIS1, MFF, and mitochondrial dynamics proteins of 49 and 51 kDa (MiD49, MiD51) (Palmer et al., 2011; Onoue et al., 2013; Otera et al., 2013; Hall et al., 2014; Atkins et al., 2016). Oxidative damage of this organelle may cause loss of ΔΨ, which leads to phosphatase and tensin homolog (PTEN)-induced putative kinase (PINK) stabilization on the MOM which recruits the E3 ubiquitin ligase parkin to ubiquitinate mitochondrial proteins and induce mitochondrial fragmentation. Excessive fragmentation of mitochondria can induce the release of cytochrome c into the cytoplasm and trigger apoptotic cell death. Balance between fusion and fission of the mitochondria has been shown to be of great importance in a wide range of cardiovascular diseases. Quality control through degradation of damaged mitochondria via mitochondrial autophagy, mitophagy, is also closely related to mitochondrial fission as these damaged/fragmented organelles can be engulfed and degraded by lysosomes through the formation of autophagolysosomes (Zaffagnini and Martens, 2016).

### Mitochondrial Turnover Through Autophagy-Mitophagy

**FIGURE 3:**
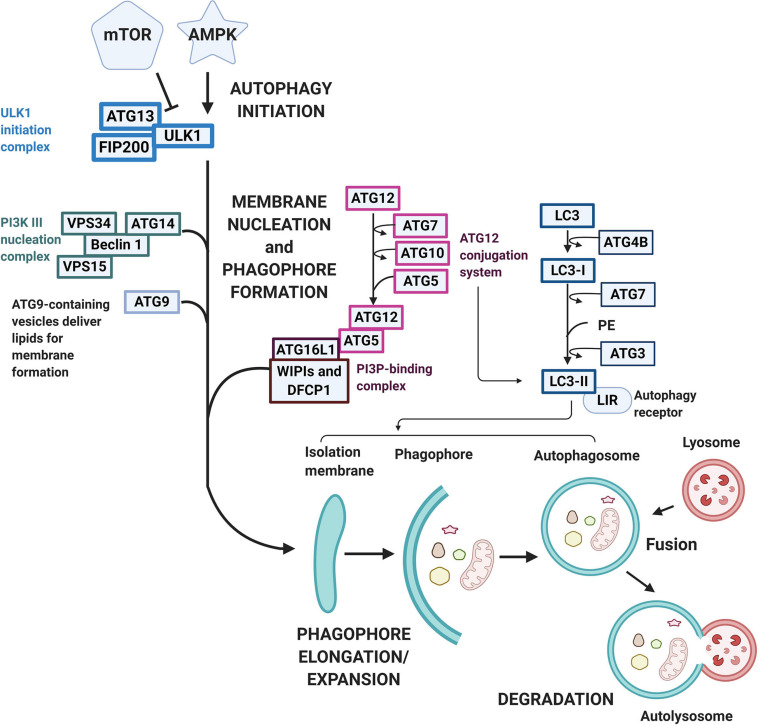
Macroautophagy pathway and signaling. Macroautophagy is activated following various stimulation under cellular stress conditions. The autophagic pathway typically includes several steps including (1) initiation, (2) phagophore formation, (3) elongation/expansion, (4) autophagosome-lysosome fusion, and (5) degradation. mTOR and AMPK regulates the initiation process with AMPK acting as an activator while mTOR acts as an inhibitor. Under cellular stress, mTOR reduces its inhibition on the ULK1-FIP200-ATG13 complex, whereas AMPK reduces mTORC activity and directs ULK-1 to the site of autophagy. The ULK-1 complex regulates the nucleation and phagophore formation process through the recruitment of VPS34 and the formation of PI3P. PI3P then contributes to the phagophore formation through the signaling of WIPI2B and DFCP to drive the phagophore formation. A double-membrane vesicle (phagophore) begins to form and elongate into an autophagosome through two ubiquitin-like conjugation cascades, the ATG5-ATG12 and the LC3 systems. The LC3 is first processed to LC3-I by ATG4B and then activated by ATG7. LC3-I is later transformed into LC3-II via ATG3 and incorporated into the limiting membrane which harbors the LIRs. The sealed degradation components are then further degraded in an acidic environment containing hydrolytic enzymes through the fusion of the matured autophagosome with lysosome to form an autolysosome.

All cells need a way to eliminate unwanted or damaged parts. The sophisticated system autophagy is crucial to prevent accumulation of toxic waste, make room for the incorporation of new elements, or reuse old building blocks (Klionsky et al., 2011; Morales et al., 2020). Three known types of autophagy have been reported, namely macroautophagy, chaperone-mediated autophagy (CMA), and microautophagy. Macroautophagy relies on the formation of autophagosomes to sequester and transport cargo to the lysosome. CMA transports unfolded proteins directly across the lysosomal membrane. Microautophagy involves the direct uptake of smaller cellular waste through invagination of the lysosomal membrane (Morales et al., 2020). Macroautophagy typically involves the degradation of large cellular components, even organelles and has been the most well-described pathway for mitochondrial turnover, called mitophagy, therefore we will be referring to macroautophagy as autophagy from now on. While non-selective autophagy is activated in responses to reduced nutrient availability or certain cellular stresses and comprise mainly the engulfment and degradation of bulk cytosolic material, selective autophagy labels specific molecules and structures destined for degradation (Parzych and Klionsky, 2013). As mentioned previously, this type of autophagy involves ubiquitin, ubiquitin-like conjugation, and activation systems. These molecular tags act as cargo recognition which ultimately expands to generate the autophagosome which then fuses with the late endosome or lysosome (Hansen et al., 2018). Both types of autophagy processes undergo the stages of (1) initiation, (2) phagophore formation, (3) elongation/expansion, (4) autophagosome-lysosome fusion, and (5) degradation (Figure 3). More than 30 autophagy-related gene (ATG) family proteins act as important mediators and orchestrate this catabolic process of damaged organelles (Parzych and Klionsky, 2013; Zaffagnini and Martens, 2016). During the initiation process, the ATG family proteins are regulated through the inhibition of the mammalian target of rapamycin (mTOR)/activation of the AMP-activated protein kinase (AMPK) pathway (Yamada and Singh, 2012). Under stress/starvation, reduction of mTOR activity releases its inhibition on the UNC-51–like kinase (ULK1) family interacting protein of 200 kD (FIP200)-ATG13 complex and thus autophagy is induced (Hara et al., 2008). Meanwhile, AMPK drives autophagy through reducing mTOR complex 1 (mTORC1) activity, while simultaneously directs the ULK1 complex to the site of autophagosome formation (Hansen et al., 2018). At the same time, with the downregulation of mTORC1, fine tune regulatory pathways such as the activation of death-associated protein 1 (DAP1), a negative regulator of autophagy, prevent the uncontrolled upregulation of autophagy (Hansen et al., 2018). The ULK1 complex regulates the recuitment of the vacuolar protein sorting 34 (VPS34) complex (Mijaljica et al., 2012). However, to form the VPS34 complex and the initiation of autophagy, *de nov*o formation of the nucleation complex via the dissociation of Beclin 1 from BCL-2 is also needed. The dissociated Beclin 1 then forms the transient VPS34 complex with ATG 14-like (ATG14L), VPS15, and the lipid kinase VPS34 to generate the functional class III phosphatidylinositol 3-kinase (PI3K) complex which is responsible for the catalyzation of phospholipid phosphatidylinositol phosphate (PIP) to phospholipid phosphatidylinositol 3-phosphate (PI3P) (Zachari and Ganley, 2017). PI3P then signals PI3P-binding proteins like WD repeat domain phosphoinositide-interacting protein 2 (WIPI2B) and double FYVE containing protein 1 (DFCP1) to the phagophore site and drives phagophore formation and eventually the formation of autophagosomes (Xie et al., 2008). For the elongation/expansion of phagophores into autophagosomes, PI3P also targets other ATG molecules, such as the recruitment of membranes through the shuttling of ATG9 and the activation of two ubiquitin-like conjugation cascades, the ATG5-ATG12 and light chain-3 (LC3) systems (Dall’Armi et al., 2013; Nascimbeni et al., 2017). ATG7 is required for catalyzing the activation and binding of ATG12 with ATG5, which subsequently interacts with ATG16 and is recruited to the site of autophagosomes. Concurrently LC3 is processed by ATG4B to expose a COOH-terminal glycine and generate LC3-1. LC3-1 is transferred to ATG3 after activation by ATG7, and transformed to the active form LC3-II with lipidation through adding phosphatidylethanolamine (PE) to its C-terminal glycine via reactions requiring the ATG5-ATG12-ATG16 complex (Dooley et al., 2014). LC3-II is then incorporated into the limiting membrane/expanding vesicle and is considered an important marker of autophagy activation. The limiting membrane eventually seals forming the autophagosome and delivers the cargo to the lysosome to be degraded. Besides the bulk autophagy process, LC3 also plays a role in the finer selective autophagy where damaged or misfolded molecules can be selectively labeled and processed. For this more efficient process to occur, recognition of the cargo and the tethering of the cargo to the autophagosome is needed (Zaffagnini and Martens, 2016). Sequestosome 1 (SQSTM1) or P62 protein in this case acts as the recognition receptor for ubiquitinated proteins and organelles. These P62 proteins contains one LC3-interacting region (LIR) motif otherwise known as the ATG8-interacting motif (AIM), which localizes to sites of autophagosome formation and binds with LC3 or other ATG8 proteins (Pankiv et al., 2007; Ichimura and Komatsu, 2010). With the tagging and recognition systems, selective cargo removal and degradation through autophagy can be achieved (Anzell et al., 2018). As the heart undergoes stressful circumstances, elimination of damaged organelles such as mitochondria plays a critical role in the balance or remodeling of the heart under pathological environments.

### Age and Mitochondria Quality Control

Aging is an irreversible biological process that typically involves the gradual deterioration of cardiac function and provokes various cardiovascular diseases through telomere shortening, genomic instability, oxidative stress, lipotoxicity, metabolic dysregulation, apoptosis, and mitophagy (Ren and Zhang, 2018; Wang S. et al., 2019; Ajoolabady et al., 2020; Onishi et al., 2021). Among these pathological etiologies, recent evidence has drawn light on and proposed that quality control through mitophagy plays a hand in the regulation of longevity and cardiovascular function in aging (Ren et al., 2017; Fernández et al., 2018; Nakamura and Yoshimori, 2018). Thai et al. (2019) reported increased parkin protein expression in aged rabbits and maximal expression in HF hearts where it was barely detectable in young animals. Associated reduction of MFN2 and DRP1 by almost 50% in such aged HF rabbit models was also observed suggesting rescuing mitochondrial fusion may preserve aging-related mitophagy defects. Additionally, β-hydroxybutyrate (β-OHB), which is one of the most abundant ketone bodies in human circulation, is shown to enhance mitophagy in young and aging myocytes, but not in HF. This could be improved, however, with the enhancement of mitochondria fusion with the TAT-MP1 glycine peptide (Thai et al., 2019). Liang et al. (2020) reported increased parkin levels with reduced DRP1−mediated mitochondrial fission and reduced autophagosome formation in the heart of aged mice further suggesting the involvement of dysregulated mitophagy in aging-related cardiovascular pathologies. Double knockout of Akt2 and AMPK enhanced age-associated suppression in mitophagy, including decreased mitophagy regulators Atg5, Atg7, Beclin 1, LC3BII-to-LC3BI ratio, PTEN-induced putative kinase 1 (Pink1), parkin, Bnip3, FundC1, and the mitochondrial biogenesis cofactor PGC-1α (Wang S. et al., 2019). These observations indicate the role of compromised autophagy and mitophagy in aging cardiovascular pathology and possible benefits of targeting proper mitochondria quality control in cardiac aging.

## Mitochondrial Inter-Talk With Nucleus

Mitochondria are essential for mammalian cells, yet they do not function as individual entities. Heavily reliant on cellular support and vice versa, the mitochondria actively signal and interact with other subcellular compartments. This inter-organelle coordination with highly organized intracellular localization is essential for cellular function and survival. To fulfill their tasks, mitochondria are in constant communication with the nucleus, endoplasmic reticulum (ER), Golgi apparatus, and other vesicular organelles (Shin et al., 2017, Shai et al., 2018; Wong et al., 2018; Lin et al., 2020). This diverse array of interplay enables multiple independent interactions without interfering with other cellular processes through compartmentalization and thus enables mitochondria to undergo multi-sided subcellular processes and metabolic cues. Inter-organelle communication between the mitochondria and other organelles especially the nucleus has been suggested to play a role in the pathophysiology of various chronic diseases.

Out of the 1,200 mitochondrial proteins required in mitochondrial biogenesis, most of the mitochondrial proteins are encoded by nuclear genes, translated and imported into the mitochondria through mitochondrial membrane translocase complexes (Pfanner et al., 2019). The biogenesis of the mitochondrial OXPHOS system is also under dual genetic control of nuclear and mitochondrial genes. Therefore, a delicate balance between nuclear and mitochondrial-encoded mitochondrial proteins is orchestrated and monitored for organelle health (Eisenberg-Bord and Schuldiner, 2017). The communication between mitochondria and the nucleus is essential for malfunctioned mitochondria to trigger compensatory nucleus responses in order to survive [64]. The nucleus controls mitochondrial gene expression and posttranslational modifications, so-called anterograde signaling; mitochondria modulate nuclear gene expression and cellular protein activity through signal transport originating from the mitochondria, termed retrograde signaling.

### Anterograde Signaling From the Nucleus to Mitochondria

A set of nuclear-encoded factors coordinately regulate mitochondrial function through anterograde signals in response to cellular environmental alteration. The process involves the expression of nuclear transcription factors and co-regulators that regulate the expression of nuclear-encoded mitochondrial proteomes, and the production of nuclear-encoded mitochondrial factors that control mtDNA gene expression (Quirós et al., 2016). All of the mitochondrial factors that activate mitochondrial transcription and translation are encoded in the nucleus, mostly nuclear respiratory factors (NRFs). There are two nuclear transcription factors, NRF1 and NRF2α also known as GA-binding protein-α (GABPα) (Miglio et al., 2009). These transcription factors, mainly NRF1, modulate the production of various nuclear-encoded mitochondrial proteins including cytochrome c, the vast majority of subunits for OXPHOS assembly, components of the mitochondrial protein import machinery, as well as proteins involved in mtDNA replication, transcription, and translation (Blesa et al., 2008). Additionally, NRF1 integrates mtDNA gene expression through direct control of the expression of important mitochondrial transcription machinery: the mitochondrial RNA polymerase (POLRMT), mitochondrial transcription factor A (TFAM), transcription specificity factors (TFB1M and TFB2M), and transcription termination factor (mTERF) (Bruni et al., 2010). During initiation of mtDNA transcription, TFAM binds to sequence specific regions of mtDNA, recruiting POLRMT to TFAM–mtDNA sequences then recruits TFB2M to accomplish promoter melting and separation of the two strands of the mtDNA thus initiating mtRNA synthesis (Kuhl et al., 2016). POLRMT also contributes to mitochondrial gene expression by regulating mtDNA replication via generation of RNA primers and interaction with TFB1M to mediate mitochondrial ribosome subunit assembly (Surovtseva and Shadel, 2013). In addition to the regulation of mtDNA transcription, TFAM is involved in many functions including mtDNA maintenance, replication, and likely also mtDNA repair (Gaspari et al., 2004; Kuhl et al., 2016). There is strong experimental evidence that the amount of TFAM directly regulates the mtDNA copy number and that mtDNA levels also reciprocally affect TFAM levels (Picca and Lezza, 2015). Additionally, there are nuclear receptors, such as peroxisome proliferator-activated receptors (PPARs) and estrogen-related receptors (ERRs), that activate the expression of nuclear-encoded mitochondrial proteins. PPARδ stimulates enzyme expressions involved in mitochondrial fatty acid oxidation while ERRs modulate the expression of nuclear-encoded mitochondrial protein in the TCA cycle, OXPHOS, and the fatty acid oxidation process (Xia et al., 2019). Estradiol acts centrally and systemically to regulate energy balance and metabolism. Sex differences in cardiovascular diseases suggest a protective role for estrogens in some diseases and the sexually dimorphic differences have been reviewed and discussed recently by Klinge (2020).

The nuclear transcription factors regulating mitochondrial protein expression require fine tuning by co-activators and co-repressors. Co-activators for stimulating mitochondrial biogenesis include the PPAR γ co-activator (PGC) family containing PGC-1α, PGC-1β, and PGC-1-related co-activator (PRC) (Gleyzer and Scarpulla, 2011). The PGCs are master regulators of mitochondrial biogenesis and play central roles in coordination and driving energy metabolism, fatty acid oxidation, gluconeogenesis, peroxisomal remodeling, and oxidative phosphorylation (Scarpulla et al., 2012). Among them, PGC-1α integrates and coordinates the activity of multiple transcription factors, including NRFs, ERRs, and PPARs and mitochondrial TFAM, which are all involved in mitochondrial biogenesis (Corona and Duchen, 2016). Other metabolism-involved transcription factors targeted by PGC-1α include Forkhead box protein O1 (FOXO1), Sterol regulatory element-binding proteins (SREBPs), Forkhead box protein A2 (FOXA2), and SRY-box transcription factor 9 (Sox9) (Gureev et al., 2019). A known co-repressor, receptor interacting protein 140 (RIP140), maintains the balance with co-activators by inhibiting mitochondrial biogenesis (Morrish and Hockenbery, 2014).

These nuclear factors and co-activators are further regulated by upstream sensors in response to changes in cellular conditions such as PGC-1α activation in cellular mitochondrial homeostasis (Fernandez-Marcos and Auwerx, 2011). In the cell, energy shortage with low ATP/AMP sensed by AMPK elevates cellular NAD^+^ levels and activates sirtuin 1 (SIRT1) to upregulate PGC-1α which leads to consequential mitochondrial biogenesis (Joo et al., 2016). Similarly, Ca^2+^ release from mitochondria during exercising and cold stress can also activates PGC-1α through promoting the mitochondrial biogenesis activation of protein kinase A (PKA) and *cAMP-response element binding* protein (CREB) (Chen et al., 2010; Gill and La Merrill, 2017; Gill et al., 2019).

### Retrograde Signaling From Mitochondria to the Nucleus

Mitochondria are a self-monitoring network, and organelle dysfunction activates retrograde signaling to the nucleus for activation of nuclear genes involved in metabolic reprogramming and stress response in restoring mitochondrial function (Liu and Butow, 2006).

#### The Mitochondrial Unfolded Protein Response (UPR^*mt*^)

A retrograde signaling pathway, the UPR^*mt*^, in which signals are activated upon impaired mitochondrial protein import, transduct to the nucleus, and induce transcription factors to regulate repair of the mitochondrial network and prevent cell death (Semenza, 2011; Shpilka and Haynes, 2018; Cavalcante et al., 2020). UPR^*mt*^ promotes a rewiring of cellular metabolism that includes the suppression of TCA and OXPHOS encoding genes potentially to relieve mitochondrial stress and enhance genes expression in glycolysis and amino acid catabolism simultaneously as alternative energy sources to promote cellular survival (Anzell et al., 2018; Pfanner et al., 2019). In addition to cellular metabolism alterations, UPR^*mt*^ increases expression of mitochondrial localized chaperones, proteases, protein import machinery in proteostasis, as well as the expression of anti-oxidative proteins in redox homeostasis in order to repair dysfunctional mitochondria. Therefore, mitochondrial working burden is decreased, damaged mitochondrial proteins are efficiently cleaved and processed, mitochondrial oxidative stress is contained, and the cellular back-up energy is maintained by cellular glycolysis to augment mitochondrial recovery (Quirós et al., 2016). Various conditions of mitochondrial dysfunction could affect mitochondrial protein import through the MOM including OXPHOS impairment, mtDNA defects, perturbation in mitochondrial translation and protein synthesis, overexpression of irreversibly misfolded mitochondrial proteins, mitochondrial proteostasis imbalance, excess ROS generation, and amino acid depletion (Qureshi et al., 2017). In *Caenorhabditis elegans*, a main messenger for the initiation of UPR^*mt*^, is the activating transcription factor associated with stress-1 (ATFS-1) which has two targeting sequences: the nuclear localization sequence (NLS) and the mitochondrial-targeting sequence (MTS). In a healthy mitochondrial network, the MTS prevails and ATFS-1 is imported into the mitochondria where it is degraded by the matrix-localized protease Lon. In the event of mitochondria defect, mitochondrial protein import is impaired and ATFS-1 is directed to the nucleus via NLS and activates UPR^*mt*^ (Wu et al., 2018). In the nucleus, ATFS-1 downregulates OXPHOS gene transcription, upregulates the expression of mitochondrial chaperones and proteases for proteostasis, and imports machineries to restore mitochondrial homeostasis (Wu et al., 2018). In mammals, the regulation of UPR^*mt*^ is not yet completely elucidated. However, evidence has shown that transcription factors C/EBP homologous protein (CHOP), *activating transcription factor 4* (ATF4), and ATF5 are involved in mammalian UPR^*mt*^ (Shpilka and Haynes, 2018). Their activation is reliant upon the eukaryotic translation initiation factor 2 subunit 1 (eIF2α) which is catalyzed by four kinases including general control non-derepressible-2 kinase (GCN2), protein kinase RNA (PKR), PKR-like endoplasmic reticulum kinase (PERK), and heme-regulated inhibitor kinase (HRI) in response to diverse cellular stresses (Teske et al., 2013; van Leeuwen and Rabouille, 2019). The activation of eIF2α results in reduced global protein synthesis and preferential translation of the transcription factors CHOP, ATF4, and ATF5 (Qureshi et al., 2017). ATF5 is thought to be orthologous to the C. elegans’ ATFS-1 and is regulated via mitochondrial protein import efficiency and other diverse forms of mitochondrial stress (Melber and Haynes, 2018). In mammals, CHOP, ATF4, and ATF5 increase the expression of mitochondrial chaperones and proteases, metabolic remodeling, the mitochondrial-protein-import-related complex, mitochondrial biogenesis related components, and anti-oxidative enzymes (SOD, glutathione synthesis machinery, and ubiquinone synthesis genes) (Qureshi et al., 2017). When perturbations occur within the mitochondrial intermembrane space, the nuclear hormone receptor estrogen receptor α (ERα) is activated in addition to CHOP, ATF4, and ATF5 to increase intermembrane space protease high-temperature requirement A2 (HTRA2) expression and also NRF1 for mitochondrial biogenesis promotion. UPR^*mt*^ also mediates global gene silencing in response to eIF2α activation probably due to the need for cutting energy expenses in anabolism under decreased TCA-OXPHOS-ATP production. The global gene silencing in UPR^*mt*^ is mediated through chromatin remodeling due to cytosolic protein LIN-65 translocation to the nucleus to induce chromatin compaction. Since the mitochondrial stress response genes need to be kept transcriptionally competent under global gene silencing, UPR^*mt*^ activates the histone lysine demethylases Jumonji C domain-containing protein -1.2 (JMJD-1.2) and JMJD-3.1 and the homeo box transcription factor defective proventriculus -1 (DVE-1) to promote an open chromatin state (Merkwirth et al., 2016; Livezey et al., 2018).

#### Other Retrograde Signaling Pathways

Besides UPR^*mt*^, there are other retrograde signals induced by mitochondrial damage. The low ATP/AMP caused by mitochondrial dysfunction activates AMPK and inhibits the mTORC1, which stimulates mitochondrial biogenesis, autophagy, and lysosomal degradation (da Cunha et al., 2015; Herzig and Shaw, 2018). The mTOR kinase pathway integrates a multitude of extracellular and intracellular cues to drive growth and proliferation. Under stress and starvation, inhibition of mTORC1 coordinates energy consumption by the mRNA translation machinery and mitochondrial energy production by stimulating synthesis of nucleus-encoded mitochondria-related proteins including TFAM, mitochondrial ribosomal proteins, and components of complexes I and V (Morita et al., 2015). Mitochondrial oxidative stress also signals the nucleus to upregulate antioxidative enzymes in nDNA through activation of transcription factors such as nuclear factor erythroid 2 related like 2 (NFE2L2) (Merry and Ristow, 2016; Hu et al., 2018). Excessive mitochondrial ROS under a hypoxic environment activates nucleus transcription factor hypoxia inducible factor 1 (HIF-1) which switches gene expression from oxidative to glycolytic metabolism. HIF-1 can also trigger mitophagy by activating the gene encoding proapoptotic BNIP3 (Zhang and Ney, 2009; Semenza, 2011). Ca^2+^ retrograde signaling occurs in the event of mitochondrial stress when the mitochondria lose their membrane potential with consequent release of Ca^2+^ into the cytoplasm. Elevated free cytosolic Ca^2+^ activates phosphatase calcineurin, which activates the transcription factors nuclear factor-κB (NF-κB) and nuclear factor of activated T cells (NFATC). The two translocate to the nucleus to promote synthesis of proteins involved in Ca^2+^ transport and storage (Liu et al., 2017). Elevated cytosolic Ca^2+^ also activates other Ca^2+^-regulated kinases, such as calcium/calmodulin-dependent protein kinase IV (CAMKIV), Ca^2+^-dependent protein kinase C, c-Jun N-terminal kinases (JNK), and p38 MAPK; which stimulate different transcription factors such as CREB, early growth response protein 1 (EGR1), cAMP-dependent transcription factor ATF2, CCAAT/enhancer-binding protein δ (CEBPδ), and CHOP to mediate mitochondrial adaptation, Ca^2+^ metabolism, glucose metabolism, and cell proliferation (Giorgi et al., 2018). Another important type of retrograde signaling is the mitochondrial involvement of the intrinsic apoptosis pathway. With the release of mitochondrial pro-apoptotic proteins into the cytosol, downstream CASP 3 executes the cleavage and destruction of subcellular structures, including the nucleus (Prokhorova et al., 2018; Tang et al., 2019).

## Association of Cardiovascular Diseases and Mitochondrial Dysfunction and Future Treatment Strategies

Mitochondria with their multitude of functions are essential in high-energy demand cardiac tissues. Here we discuss three common cardiovascular diseases (HTN, Ischemic heart disease, and diabetic cardiomyopathy) and their relationship to mitochondrial dysfunction. Apart from these diseases, mitochondrial genetic haplotypes are also associated with different pathological changes in cardiovascular diseases. Since the discovery of these mitochondrial-related pathologies, mitochondrial-targeting therapeutic treatments have emerged.

### Hypertension

HTN has been considered crucial in the pathogenesis of many cardiovascular diseases. Both inflammatory damage and endothelial dysfunction of vascular structure as well as activation of the sympathetic nervous system have been alluded to have a hand in the pathogenesis of HTN (Lahera et al., 2017; Morales et al., 2020). Additionally, mitochondria dysfunction has been suggested to play a role in the pathophysiology of HTN (Barki-Harrington et al., 2004). Angiotensin (Ang) II is an important stimulus for HTN and its effects on mitochondrial function have been shown to be through the elevation of mitochondrial oxidative damage, decreases in endothelial nitric oxide (NO^.^) bioavailability, and the induction of vascular oxidative stress. Ang II causes mitochondria dysfunction through a protein kinase C dependent pathway leading to NADPH oxidase (NOX) activation and the formation of excess ROS such as O_2_^•–^ and H_2_O_2_ after a reaction of SOD and peroxynitrite (ONOO^–^) in the presence of NO, respectively (Iuchi et al., 2003; Doughan et al., 2008). Doughan et al. (2008) reported that the deleterious effect that Ang II has on mitochondria in bovine aortic endothelial cells leads to the generation of a virtuous cycle involving the increase of mitochondrial H_2_O_2_ production, the activation of cellular NOX, the increase of intracellular O_2_^•–^ production, and diminishing NO^.^ bioavailability which eventually contributes to endothelial dysfunction and activation of apoptotic signaling (Davidson, 2010). Recently, mitochondria dynamics and quality control have been shown to be involved in the process of endothelial dysfunction. Lugus et al. (2011) have shown that with the downregulation of MFN1, the major mitochondrial fusion regulator lowers angiogenic responses to vascular endothelia growth factor (VEGF) and the activation of endothelial nitric oxide synthase (eNOS) in cultured endothelial cells. Knockout of either MFN1 or MFN2 resulted in the disarray of the mitochondrial homeostasis and the decrease of mitochondria ΔΨ through the blunting of VEGF signaling pathways associated with oxidative metabolism (Lugus et al., 2011). Both N^*G*^-nitro-L-arginine methyl ester (L-NAME) treated endothelial cellular and Sprague Dawley rat models have suggested that under eNOS dysfunction, mitochondria tend to shift away from fusion and that excessive fission may be an underlying cause of endothelial cell dysfunction in postischemic hearts (Giedt et al., 2012; Miller et al., 2013). The finding that genetic variation in the PARK2 gene, which encodes the important mitophagy regulator parkin, is significantly associated with HTN in both Nigerian and Korean populations further supports the relationship of mitochondrial quality control and susceptibility to HTN (Tayo Bamidele et al., 2009; Jin et al., 2011). The protective effect of mitophagy on the development of HTN has been shown, as overexpression of cathepsin S and enhanced ATG5-mediated mitophagy contributes to the decrease of both ROS production and NF-κB-related inflammatory responses using an Ang II-induced HTN mice model (Pan et al., 2012; Zhao et al., 2014). At the same time, ER stress influences mitochondria through specialized complexes through the ER-associated mitochondria membrane and the exchanges of Ca^2+^, lipids, metabolites, and signaling molecules (Young, 2017). Inter-organelle actions of the ER leading to activation of cytosolic UPR can also cause the increase in inflammation, ROS production, and apoptosis (Sharkey et al., 2009; Santos et al., 2013). Adipocyte-related ER stress in obese subjects shows an increase of the ER chaperone glucose-regulated protein 78 (GRP78), while activating UPR signals resulting in the turning on of transcription factor 4, CHOP, and Bax in an obese ovine-related HTN model induced by increased food intake and reduced activity (Sharkey et al., 2009; Santos et al., 2013). CNS activation of UPR might also be a mediator of HTN via changes in synaptic transmission, neuronal signaling, and autonomic overaction (Young, 2017). Indeed, when the ER stress inducer thapsigargin, which causes UPR activation by perturbing cellular Ca^2+^, is applied to the brain lateral cerebral ventricle in mice it results in elevation in arterial blood pressure (Bravo et al., 2012; Young et al., 2012).

In additional to endothelial damage, HTN also causes structural alterations to the mitochondria of the cardiomyocyte, including decreased mitochondria mass and density, mitochondria swelling, and cristae remodeling (Ritz and Berrut, 2005; Eirin et al., 2014). Decrease in mitochondria size and density combined with osmotic swelling of the mitochondria lead to a gradual compromise of oxidative capacity (Ritz and Berrut, 2005). HTN was found to be associated with loss of cardiolipin, a phospholipid required for proper cristae formation, which can further threaten the stability of the ETC complexes (Cogliati et al., 2013). Lack of cardiolipin may also result in dysfunctional mitochondria dynamics, the opening of mPTP, the releasing of cytochrome C, and triggering apoptosis (Osman et al., 2011). Inhibition of mitochondria fission with the DRP1 inhibitor prevents cardiomyocytes apoptosis in spontaneous hypertensive rat models (Qi et al., 2018). The same group also found that Ang II treatment of cultured neonatal rat cardiomyocytes elevated DRP1 expression and enhanced mitochondrial fission as well as cellular apoptosis. This Ang II-induced hypertensive cardiomyopathy was suggested to be sirtuin 1(Sirt1)/P53/DRP1-dependent (Qi et al., 2018). HTN enhanced excessive ROS production can damage DNA and activate the poly (ADP-ribose) polymerase (PARP) enzyme (Dizdaroglu and Jaruga, 2012; Alemasova and Lavrik, 2019). Activated PARP enzyme binds to the damaged DNA region via cleaving NAD^+^, thus depleting NAD^+^ reserves and resulting in programmed cell death (Ordog et al., 2021). Ordog et al. (2021) that showed PARP-inhibition prevented DRP1 expression and increased fusion proteins while preserving mitochondria structure and function in cardiomyocytes. These recent findings provide valuable clues to the fact that multi-functional pathways may aid in the development of HTN-related cardiovascular complications where mitochondria quality control through mitophagy and inter-organelle inter-talk between mitochondria and ER plays a pivotal role.

### Ischemic Heart Disease

Pathological cardiac conditions resulting from ischemic cardiac injuries and occlusion of a coronary vessel induces a cascade of tissue hypoxia and cellular ATP depletion (Peoples et al., 2019). A series of events may contribute to this process of ischemic and reperfusion damage to cardiac tissues with impairment of mitochondrial integrity being central to this process, including the generation of ROS, opening of the mitochondrial permeability transition pore (mPTP), and activation of intrinsic apoptosis (Kulek et al., 2020). Under these drastic changes in nutrient and oxygen availabilities, a decrease in oxidative phosphorylation results in a decrease in cellular ATP, and a loss of mitochondrial membrane potential (Murphy and Steenbergen, 2008). With the I/R injuries and cytosolic changes of the cardiomyocytes, lowering of intracellular pH due to lactate accumulation results in cytosolic Ca^2+^ overload (Murphy and Steenbergen, 2008). High cytosolic Ca^2+^ in turn leads to an overload of mitochondrial Ca^2+^, which combined with unregulated ROS production from IRI, brings about the opening of the mPTP (Halestrap and Pasdois, 2009). The act of mPTP opening gives rise to the permeabilization of the MIM, thus causing mitochondrial depolarization, loss of membrane potential, swelling, rupture, and ultimately cell death (Halestrap and Pasdois, 2009; Kwong and Molkentin, 2015). Therapeutic strategies targeting to prevent the opening of mPTP through immunosuppressant cyclosporin-A (CsA) and sanglifehrin-A (SFA) have been shown to protect the myocardium from IRI in preclinical studies (Hausenloy et al., 2003; Hausenloy and Yellon, 2003). Hausenloy et al. (2002, 2003) have shown that CsA and SFA significantly decreases myocardial infarct size using isolated rat heart models subjected to 35 min of ischemia and 120 min of reperfusion. However, Cung et al. (2015) found that intravenous cyclosporine did not improve clinical outcomes in patients with anterior STEMI who had been referred for primary percutaneous coronary intervention, more than those with placebo in the Does Cyclosporine Improve Clinical Outcome in ST Elevation Myocardial Infarction Patients (CIRCUS) trial. Other inhibitors of mPTP such as 3,5-Seco-4-nor-cholestan-5-one oxime-3-ol (TRO40303) and elamipretide (MTP-131) also did not show any effect on limiting ischemic injury as can be seen in the MITOCARE and EMBRACE STEMI studies (Atar et al., 2015; Gibson et al., 2016). Facing difficulties in direct inhibition of mPTP, halting mPTP through targeting activators such as ROS may be a novel therapeutic strategy. Adlam et al. (2005) and Ribeiro Junior et al. (2018) revealed promising results in rat I/R models with mitochondria-targeted antioxidants such as mitoQ. As mentioned previously, mitochondrial quality control through mitophagy is crucial for proper maintenance mitochondrial function in cells under stress. In most mammalian tissues, mitochondria adapts to stress through a series mitochondrial dynamic processes including fission and fusion (Kulek et al., 2020). However, it has been reported that in adult cardiomyocytes, mitochondria dynamics are scarce with slow mobility and little morphology change (Song and Dorn, 2015). Thus, the sequestration and mitophagic removal of this damaged organelle is paramount for maintaining mitochondria homeostasis and relieving IRI damage (Song and Dorn, 2015). Therefore, quality control of mitochondria via mitophagy under I/R stress can be on a knife’s edge, as excessive activation of mitophagy may result in disastrous loss of mitochondria leading to cellular apoptosis (Kulek et al., 2020). In the meanwhile, insufficient mitophagic activation in response to cellular stress may give rise to the accumulation of dysfunctional mitochondria (Kulek et al., 2020). Factors known for inducing autophagy like Beclin 1, BCL2/adenovirus E1B 19 kDa protein-interacting protein 3 (BNIP3), and FUN14 domain containing 1 (FUNDC1), and anti-autophagic factors like BCL2 have been proposed to play roles during IRI (Georgakopoulos et al., 2017; Zhang et al., 2017). Matsui et al. (2007) found that for *Becn1*^+/–^ mice cardiomyocytes, autophagy is protective during cardiac ischemia, but is detrimental during reperfusion. However, a protective effect through inhibition of *Bnip3*-related autophagy has been demonstrated in studies by Diwan et al. (2007), which knocked out this pro-mitophagic regulator *Bnip3* and preserved left ventricle systolic performance and diminished left ventricle dilation in *Bnip3*^–/–^ mice under I/R-induced myocardial damage. Though this myocardial salvage effect may be due to inhibition of apoptosis via caspase-dependent apoptosis and mPTP opening as is later supported by Matsui et al. (2007) and Bravo-San Pedro et al. (2017). Ischemia can also cause nuclear receptor subfamily 4 group A member 1 (NR4A1) expression, which in turn activates serine/threonine kinase casein kinase 2α (CK2α) and promotes CK2-related phosphorylation of MFF and FUNDC1 (Zhou et al., 2018a). Phosphorylated activation of MFF leads to fatal mitochondrial fission in addition to the inactivation of FUNDC1-associated protective mitophagy (Zhou et al., 2018a). Zhou et al. (2018a) found that in NR4A1 knockout mice, the loss of CK2 restores FUNDC1-mediated mitophagy further providing a survival advantage to cardiac myocytes in response to I/R stress. Zhang et al. also showed that this mitophagy receptor FUNDC1 interacts with LC3 to mediate mitophagy in response to hypoxia in cultured cardiomyocytes. Using a *Fundc1*−/− mouse model, these authors also provide evidence that FUNDC1-mediated mitophagy regulates mitochondrial quality control in vivo and plays a crucial role in maintaining functional integrity in platelet activation and reduces I/R-induced heart injury (Zhang et al., 2016, 2017). Therefore, how to rightly facilitate and balance mitophagy to protect the heart under I/R stress remains to be elucidated by therapeutic strategies, and is therefore a new topic for investigation.

### Diabetic Cardiomyopathy

Patients with T2DM are vulnerable to vascular disease, and heart failure is the main cause of death in the diabetic population (Missiroli et al., 2018). Additionally, prevalence of T2DM is reaching pandemic proportions (Mozaffarian et al., 2015), and mortality and morbidity related to its macrovascular and microvascular complications have steadily been on the rise (Kearney, 2015; Kochanek et al., 2016). These vascular complications originate from insulin-resistance–related endothelial dysfunction, leading to higher risks of cardiovascular disease-related mortality in DM patients (Ting et al., 1996; Pieper et al., 1997). Furthermore, clinical outcomes associated with heart failure are considerably worse with an increase of mortality for patients with DM than for those without (Jia et al., 2018). A recent term “diabetic cardiomyopathy” is used to define the existence of abnormal myocardial structure and performance in the absence of other cardiac risk factors, such as coronary artery disease, HTN, and significant valvular disease, in individuals with DM (Yancy et al., 2013; Jia et al., 2018). Dysfunctional mitochondria have been proposed as a common pathologic mechanism of diabetic cardiac dysfunction (Schilling, 2015). The cardiomyocyte utilizes different substrates simultaneously for energy production with up to 60–70% ATP production from the mitochondrial oxidation of fatty acids and a lesser extent of 30–40% ATP from glucose, lactate, and other substrates (Bugger and Abel, 2010). However, in a diabetic heart, glucose utilization is greatly diminished due to insulin resistance, impaired pyruvate dehydrogenase activity, and reduced glucose transporter translocation (Heather and Clarke, 2011; Heather et al., 2013). Therefore, diabetic cardiomyocytes almost exclusively rely on mitochondrial fatty acid oxidation for ATP generation (Wright et al., 2009). This produces greater levels of oxidative stress and potentially provokes mitochondrial dysfunction, the release of pro-apoptotic factors such as cytochrome *C*, apoptosis inducing factor, and Smac/DIABLO, and altered mitochondrial Ca^2+^ handling which eventually lead to the death of cardiomyocytes (Brown et al., 2017). Mitochondria are important regulators in the cellular fuel choice of fatty acid or glucose metabolism under the changing metabolic environment. For example, fatty acid uptake is enhanced in diabetic cardiomyocytes which cause the activation of PPAR-α, an activator of genes involved in fatty acid uptake and β-oxidation. PPAR-α, on the other hand, suppresses the expressions of genes associated with the TCA and mitochondrial OXPHOS (Lee et al., 2017). Supporting this, Finck et al. reported that the activation of cardiac PPAR-α overexpression results in reciprocal repression enzymes involved in glucose uptake and utilization pathways (Finck et al., 2002). Ferreira et al. (2013) also showed that mitochondria from the heart of an STZ-induced type 1 DM mouse model presented lower OXPHOS activity and downregulation of mitochondrial transcription factor TFAM (Jiang et al., 2014; Khuchua et al., 2018). The increase of fatty acid β-oxidation augments delivery of electrons to the mitochondrial electron transport chain, causing elevated mitochondrial inner membrane potential, which stimulates mitochondrial ROS production (Liesa and Shirihai, 2013). Excessive fatty acid uptake in diabetic hearts reduce the expressions of genes involved in mitochondrial OXPHOS (Lee, 2020). Supporting these, Petersen et al. (2004) have also reported that insulin resistance in the skeletal muscle of insulin-resistant offspring of patients with T2DM is associated with the dysregulation of intra-myocellular fatty acid metabolism, possibly because of an inherited defect in mitochondrial OXPHOS. In a normal functioning cardiomyocyte, mitochondria oxygen consumption is generally tightly coupled to ATP synthesis via the ETC chain. However, in a diabetic heart there is increased proton bypass of the final ATPase to reenter the mitochondrial matrix known as mitochondrial uncoupling which causes the decrease in ATP production efficiency (Krauss et al., 2005; Demine et al., 2019). This uncoupling causes oxidative stress and is mostly noted at the mitochondrial complex I and III. Moreover, mitochondrial OXPHOS complexes were noted to be suppressed in a diabetic heart associated with increased ROS and nitrogen free radical production leading to cell death in human myocardial samples and a streptozotocin (STZ)-induced type 1 DM model (Sivitz and Yorek, 2010; Raza et al., 2011). Of note, abnormal metabolism developed in diabetic pathogenesis-induced altered mitochondrial morphology through imbalanced mitochondrial dynamic regulators such as MFN1, MFN2, OPA1, and DRP1 in various tissues (Williams and Caino, 2018). Particularly, T2DM is associated with reduced MFN2 expression, impaired mitochondrial fusion, fragmented mitochondria networks which are related to depressed OXPHOS, and glucose intolerance (Mootha et al., 2003). Montaigne et al. (2014) also demonstrated that MFN1 was decreased with mitochondrial fragmentation in relation to myocardium contractile dysfunction in human T2DM subjects (Liesa and Shirihai, 2013). Lack of fusion machinery has been noted in diabetic cardiomyocytes and Parra et al. (2014) demonstrated in rat cardiomyocytes that the fusion machinery OPA1 could be stimulated by insulin administration and promote mitochondrial fusion through a mechanism involving the Akt-mTOR-NFκB signaling pathway.

It is generally supported that alteration of mitochondrial architecture activates mitochondrial quality control mechanisms such as autophagy. Autophagy in the heart is intricately regulated and both protective or detrimental results have been reported. It is generally suggested that autophagy is suppressed in type 1 and induced in T2DM (Mellor et al., 2011). Using both transgenic and STZ-induced type 1 DM mouse models, Xu et al. (2013) reported markedly decreased autophagy core components including LC3, ATG5, and ATG12 which demonstrate inhibited autophagic flux in the cardiomyocytes of type 1 DM animals. This study also demonstrated that restoration of autophagy to transgenic mice further exacerbated diabetic cardiac injury; while cardiac damage in WT diabetic mice was substantially attenuated in Beclin 1 or ATG16L1-deficient (beclin 1^+/–^ or ATG16L1-HM) mice. Improved glucose blood levels, reduced free fatty acids, and triglycerides were observed with the suppression of autophagy regulators Beclin 1 or ATG16L1 in STZ and OVE26 diabetic mice (Xu et al., 2013). Also noted in the study is that reduced autophagy was associated with increased expression and lysosomal localization of Rab9, which is involved in the Rab9-dependent alternative autophagic pathway (Nishida et al., 2009). Together, this study suggests that the inhibition of autophagy was an adaptive response to limit cardiac dysfunction in type 1 DM, with upregulation of alternative autophagy and improved mitophagy (Xu et al., 2013). On the other hand, Kanamori et al. (2015) demonstrated in STZ-induced type 1 DM mice that induced autophagic activity was significantly associated with impaired diastolic function as documented with increasing LC3-II, SQSTM1/p62, cathepsin D, and an abundance of autophagic vacuoles and lysosomes detected via electron-microscopy.

A distinct bioenergetic impairment of heart mitochondrial subpopulations in diabetic cardiomyopathy is associated with obesity (Lai et al., 2020). Reduced cardiac efficiency, decreased mitochondrial energetics, enhanced oxidative damage, increased fatty acid oxidation, and change of heart substrate usage is found in both obese and diabetic patients (Kasper et al., 1992; Boudina et al., 2007; Boden, 2008; Peterson and Gropler, 2010). Toledo et al. (2006) showed in a study with 11 obese insulin-resistant human participants that mitochondria morphological alterations of skeletal muscles are associated with improvements in insulin resistance and weight loss. Tong et al. (2019) recently reported that mitophagy was associated with obesity in an Atg7-dependent manner. This group demonstrated the elevation of LC3-II localization to mitochondria after 3 weeks of high fat feeding. Thomas et al. (2019) also showed a decrease in cardiac parkin, a crucial protein of mitophagy in obese mice which suggests parkin-dependent mitophagy may contribute to obesity-related cardiovascular risk. All of the above suggest that both DM and obesity cardiomyopathy are pathologically associated with mitochondrial function maintenance.

### The Association of Mitochondrial Genetic Variations With Cardiovascular Disease

As have been described in the mitochondria biology section, the mitochondrion plays a pivotal role in cell physiology not only in supplying energy to the cells but is also involved in various important cellular functions. Variations of mtDNA can potentially alter mitochondrial function and thus possibly links to clinical disease (Ma et al., 2014). The association between mtDNA variations and cardiovascular risks have been well-described. For example, the pathological mtDNA A3243G mutation causes mitochondrial encephalomyopathy, lactic acidosis and stroke-like episodes syndrome (MELAS), and maternal inherited diabetes mellitus (Liou et al., 2000, 2003, 2004; Chen et al., 2004). Both the coding and control regions of mtDNA play roles in the generation of diabetes. An mtDNA variant commonly found in the general population, the T-to-C transition at np 16189, has been shown to be positively correlated with blood fasting insulin and T2DM in a population-based case-control study in Cambridgeshire, United Kingdom (Poulton et al., 2002). Based on previous studies, this mtDNA T16189C variant is associated with increased oxidative damage, altered antioxidative status in T2DM patients, metabolic syndrome, higher fasting insulin concentration, insulin resistance index, and lacunar cerebral infarction in the Asian population (Lin et al., 2005; Liou et al., 2007, 2010; Tiao et al., 2009; Wang et al., 2009). Tanaka et al. (2007) studied genotypes for 25 polymorphisms in the coding region of the mitochondrial genome and revealed that haplogroup N9a is significantly associated with resistance to the metabolic syndrome in women. Our group has reported that the haplotype B4 (carrying the T16189C variant) is associated with T2DM (odds ratio [OR], 1.54 [95% CI 1.18–2.02]; *P* < 0.001) in the Chinese population, whereas subjects harboring haplogroup D4 have borderline resistance against DM generation (0.68 [0.49–0.94]; *P* = 0.02) (Liou et al., 2010). Recently, a patient study with 830 Taiwanese ischemic stroke patients and 966 normal controls revealed an association between mitochondrial haplogroup F1 and risk of ischemic stroke (OR 1.72:1.27–2.34, *p* = 0.001) (Tsai et al., 2020). In the same study, with the usage of a hybrid technique, Tsai et al. (2020) demonstrated that mitochondrial haplogroup F1 cybrids were associated with decreased oxygen consumption, higher mitochondrial ROS production, and lower mitochondrial membrane potential. These cybrids were also noted to be prone to inflammation, with increased expression of several inflammatory cytokines. Recently Wei et al. (2021) analyzed 996 mtDNA components in the peripheral blood of patients with cardiovascular disease and detected strong associations between the patients’ clinical characteristics and both mtDNA copy number and rare mtDNA variants. All these data support the involvement of mitochondrial genetic variation in the pathogenesis of T2DM and associated vascular diseases in both clinical and functional studies.

### Therapeutic Strategies Targeting Mitochondria Protection in Cardiovascular Diseases

Mitochondrial dysfunction may lead to the pathology of many common disorders, including I/R injury (Marin et al., 2021), heart failure, metabolic disease (for example HTN, insulin resistance, and obesity) (Jha et al., 2017; Tavallaie et al., 2020), and neurodegeneration (Reeve et al., 2016; Riley and Tait, 2020). Several strategies aimed at therapeutically restoring mitochondrial function are emerging, including behavioral interventions of diet or exercise (Ascensao et al., 2007; Miller et al., 2020), exposure to hypoxia, and stem cell therapies (Lin T.K. et al., 2019). Gene therapies to correct a defective gene and degrade mutated mtDNA are also developing for traditional mitochondria diseases caused by pathologic mutation in mtDNA (Slone and Huang, 2020). Agents have recently been developed specifically for the treatment of mitochondrial dysfunction including antioxidants such as coenzyme Q10 (CoQ10), protective compounds targeting the mitochondria such as MitoQ, agents to replenish NAD^+^ pools such as nicotinamide mononucleotide (NMN) and Bendavia (SS31), and the inhibitor of the mitochondrial permeability transition pore (mPTP) cyclosporin A (CsA) (Scatena et al., 2007; Murphy and Hartley, 2018). Apart from these strategies, some specific medications already proved and used widely for clinical conditions have been suggested to provide an additional mitochondrial protection effect and their clinical use can potentially be expanded for the purpose of prevention and treatment of cardiovascular diseases.

### Repurposing Drugs to Target Mitochondrial-Related Cardiovascular Diseases

Given the high failure rate in clinical trial and costly and slow pace of new drug development, repurposing “old” drugs to treat both common and rare diseases is increasingly becoming an attractive concept because it involves the use of safety-proven compounds, with potentially lower overall development costs and shorter development timelines (Pushpakom et al., 2019). Experimental approaches have also identified repurposable candidate drugs targeting the mitochondria for common pathologies (Yoshida, 2017; Bell et al., 2018; Pellattiero and Scorrano, 2018). For example, metolazone, a diuretic primarily used to treat congestive heart failure and high blood pressure, was found to upregulate protective cellular chaperone, heat shock protein-6, and induce UPR^*mt*^, a mitochondrial stress response shown to promote longevity in model organisms (Ito et al., 2021). Ito et al. (2021) also demonstrated that metolazone extended the lifespan of Caenorhabditis elegans and specifically induced the expression of mitochondrial chaperones for UPR^*mt*^ in the HeLa cell line.

Numerous prescribed anti-diabetic agents worldwide have been found to possess cardiovascular benefits through their original glucose lowering effect. Beside, some anti-diabetic drugs have recently been proposed to be associated with mitochondrial function regulation (Foretz et al., 2014). For example, metformin, the most commonly prescribed anti-diabetic agent was shown to have the advantage of counteracting cardiovascular complications associated with diabetes in the large cohort United Kingdom Prospective Diabetic Study (UKPDS) (Bailey and Grant, 1998). Studies further show metformin to improve mitochondrial respiratory activity through paradoxical mechanisms, through reducing cellular oxygen consumption via inhibiting mitochondrial complex 1 activity at high levels of metformin or by activation of AMPK (Wang Y. et al., 2019). Another T2DM drug that have been shown to possess mitochondrial regulatory mechanisms are the sodium glucose cotransporter 2 (SGLT2) inhibitors. In three cardiovascular outcome trials: the EMPA-REG OUTCOME trial (7064 participants) (Zinman et al., 2015; Fitchett et al., 2019), CANVAS Program (4330 participants) (Neal et al., 2017), and DECLARE TIMI 58 trial (17,190 participants) (Wiviott et al., 2019) SGLT2 inhibitors have been shown to reduce cardiovascular events including mortality and hospitalization for heart failure, in patients with T2DM. The recent DAPA-HF trial additionally demonstrated SGLT2 inhibitor to reduce major outcomes in patients with established heart failure with a reduced ejection fraction (HfrEF), regardless of the presence of diabetes (McMurray et al., 2019). Two other trials currently evaluating the effects of SGLT2 inhibitors in patients with established heart failure with a preserved ejection fraction (HFpEF) regardless of the presence of diabetes are the EMPEROR-Preserved trial [NCT03057951], and the DELIVER trial [NCT03619213] (Anker et al., 2019). Salutary mechanisms of SGLT2 inhibitors in cardiomyocytes through mitochondrial function-mediated beneficial effects have been demonstrated by Takagi et al. (2018), who reported SGLT 2 inhibitors to alleviate mitochondrial dysfunction via restoration of dynamic proteins to normal values in OPA1, Mfn1, Mfn2, Fis1, and Drp1 in high-fat diet rat models. Another mechanisms of SGLT 2 inhibitor on mitochondria was through activating AMPK and suppressing mitochondrial fission (Zhou et al., 2018b). The other T2DM drug is the glucagon-like peptide-1 receptors agonists (GLP-1RA). GLP-1 receptors are found on human cardiac tissue (Anagnostis et al., 2011), and are found to have cytoprotective actions in the heart (Baggio et al., 2018). In the LEADER trial and SUSTAIN-6 trial, GLP-1RA also showed effects of reducing the rates of myocardial infarction, cardiovascular deaths, and stroke (Marso et al., 2016; Aroda et al., 2017; Nauck et al., 2017). GLP-1 RA additionally improved vasodilation and significantly reduced systolic blood pressure (Plutzky, 2011). Tomas et al. (2011) show GLP-1-derived nonapeptide GLP-1(28–36) amide targets to mitochondria and suppresses the OXPHOS process and oxidative stress (Ussher and Drucker, 2012). Chang et al. (2018) demonstrated that GLP-1 analog decreases mitochondrial morphological abnormalities, reduces oxidative stress, enhances ATP synthesis, mitochondrial ATPase activity and ΔΨ, decreases mitochondrial calcium overload and inhibits the opening of mPTP in an cellular model of I/R injury. Therefore, demonstrating GLP-1 analogue cardioprotective effects. In myocardial infarction mice, circulating GLP−1 concentrations were markedly elevated with association of increased AMPK activity which stimulated the mitochondrial respiratory capacity of non−infarcted tissue areas considered as a compensatory protective mechanism (Diebold et al., 2018). The above support the potential of drug repurposing targeting mitochondrial function modulation and implicate the dual effects of mitochondrial-protective benefits additional to their original pharmacological mechanisms for the treatment of cardiovascular disease.

## Perspective

Proper mitochondria functioning is fundamental for cellular health and of extreme importance in high-energy demand tissues. The quality control of the organelle is crucial for the removal of damaged mitochondria and maintaining homeostasis to preserve cardiac function under pathological conditions. As has been shown above, in the pathogenesis of cardiovascular diseases such as HTN, ischemic heart disease, and T2DM, mitochondria dysfunction plays a potential role in not only cellular injuries but also the progression of these diseases. Thus, providing and sustaining a healthy mitochondrial network through mitochondrial quality control can be pivotal for the outcome of the cardiac injury under various pathologic stresses. Recent development of mitochondria targeting agents have provided pharmacological means of alleviating these mitochondrial functional impairments. Old drugs have also been approached to search for potential underlying mitochondrial targeting mechanisms. These therapeutic measures present alternative or coadministration pharmacological choices and bring innovation, challenge, and hope to future remedies of common pathological pathways of cardiovascular disease. Rising evidence has given us an insight on how reducing oxidative stress, facilitating mitophagy, and even inter-organelle interaction can provide cardioprotective effects. Targeting and modulating these protective pathways may throw new light on the treatment of cardiovascular disease in the future.

## Author Contributions

K-LL contributed to the concept generation, data interpretation, drafting of the manuscript, and graphic drawing. S-DC contributed to the concept generation, data interpretation, and drafting of the manuscript. K-JL contributed to the concept generation, graphic drawing, and drafting of the manuscript. C-WL, Y-CC, P-WW, and J-HC contributed to the concept generation, data interpretation, and approval of the article. T-KL contributed to concept generation, data interpretation, graphic drawing, drafting of the manuscript, and approval of the article. All authors contributed to the article and approved the submitted version.

## Conflict of Interest

The authors declare that the research was conducted in the absence of any commercial or financial relationships that could be construed as a potential conflict of interest.

## Glossary

**Table:**

ATP	Adenosine triphosphate	MiD49	Mitochondrial dynamics protein of 49
AIM	ATG8-interacting motif	MiD51	Mitochondrial dynamics protein of 51
AMPK	AMP-activated protein kinase	MIM	Mitochondrial inner membrane
Ang	Angiotensin	MOM	Mitochondrial outer membrane
APAF -1	Apoptotic protease activating factor-1	MOMP	Mitochondrial outer membrane permeabilization
ATFS-1	Activating transcription factor associated with stress-1	mPTP	Mitochondrial permeability transition pore
ATF	*Activating transcription factor*	Msr	Methionine sulfide reductase
ATG	Autophagy related gene	mtDNA	Mitochondrial DNA
ATG14L	Autophagy related gene-like	mTERF	Mitochondria transcription termination factor
BAK	BCL-2 antagonist or killer	mTOR	Mammalian target of rapamycin
BAX	BCL-2-associated X protein	mTORC1	mTOR complex 1
BCL-2	B-cell lymphoma 2	MTS	Mitochondrial-targeting sequence
BCL-xL	B-cell lymphoma-extra large	NFE2L2	Nuclear factor erythroid 2 related like 2
Becn1	Beclin 1	NFATC	Nuclear factor of activated T cells
BNIP3	BCL2/adenovirus E1B 19 kDa protein-interacting protein 3	NF-κB	nuclear factor-κB
Ca^2+^	Calcium	NLS	Nuclear localization sequence
CAMKIV	Calcium/calmodulin-dependent protein kinase IV	NOX	NADPH oxidase
C/EBP	CCAAT/enhancer-binding protein	NRF	Nuclear respiratory factor
CHOP	C/EBP homologous protein	NR4A1	Nuclear receptor subfamily 4 group A member 1
CK2	Casein kinase 2	ONOO^–^	Peroxynitrite
CMA	Chaperone-mediated autophagy	OPA1	Optic atrophy protein 1
CREB	*cAMP-response element binding* protein	OXPHOS	Oxidative-phosphorylation
CsA	Cyclosporin-A	O_2_^.–^	Superoxide
DAP 1	Death-associated protein 1	PE	Phosphatidylethanolamine
DFCP-1	Double FYVE containing protein 1	PRC	PGC-1-related co-activator
DM	Diabetes mellitus	PINK	Phosphatase and tensin homolog (PTEN)-induced putative kinase
DRP1	Dynamin-related protein	PIP	Phospholipid phosphatidylinositol phosphate
DVE-1	Defective proventriculus -1	PI3K	Phosphatidylinositol 3-kinase
EGR1	Early growth response protein 1	PI3P	Phosphatidylinositol 3-phosphate
eNOS	Endothelial nitric oxide synthase	PKA	Protein kinase A
ER	Endoplasmic reticulum	POLRMT	Mitochondrial RNA polymerase
ERAD	ER-associated degradation	PPARs	Peroxisome proliferator-activated receptors
ERR	Estrogen-related receptors	PGC	PPAR γ coactivator
ERα	Estrogen receptor α	RIP140	Receptor interacting protein 140
ETC	Electron transport chain	ROS	Reactive oxygen species
GABPα	GA-binding protein-α	SFA	Sanglifehrin-A
GLP-1RA	Glucagon-like peptide-1 receptors agonists	SGLT2	Sodium glucose cotransporter 2
FIS1	Fission 1	SIRT1	Sirtuin 1
FOXA-2	Forkhead box protein A2	SMAC	Second mitochondria-derived activator of caspases
FOXO1	Forkhead box protein O1	Sox9	SRY-Box Transcription Factor 9
FUNDC1	FUN14 domain containing 1	SQSTM1	Sequestosome 1
GTPases	Guanosine triphosphatase	SREBPs	Sterol regulatory element-binding proteins
GRP78	Glucose-regulated protein 78	TCA	Tricarboxylic acid cycle
HIF-1	Hypoxia inducible factor 1	TFAM	Transcription factor A, mitochondrial
HTN	Hypertension	TFB1M	Transcription Factor B1, Mitochondria
H_2_O_2_	Hydrogen peroxide	TFB2M	Transcription Factor B2, Mitochondria
IMS	Intermembrane space	TNF	Tumor necrosis factor
I/R	Ischemia/reperfusion	TNFR1	Tumor necrosis factor 1
IRI	Ischemia/reperfusion injury	Trail	TNF-related apoptosis-inducing ligand
JMJD	Jumonji C domain-containing protein	UPS	Ubiquitin/26S proteasome system
JNK	c-Jun N-terminal kinases	ULK-1	UNC-51–like kinase
LC3	Light chain-3	VPS34	Vacuolar protein sorting 34
LIR	LC3-interacting region	VEGF	Vascular endothelia growth factor
L-NAME	N^*G*^-nitro-L-arginine methyl ester	WIPI2B	WD repeat domain phosphoinositide-interacting protein 2
MCL-1	Myeloid cell leukemia 1	XIAP	X-linked inhibitor of apoptosis protein
MFF	Mitochondrial fission factor	ΔΨ	Mitochondrial membrane potential
MFN1	Mitofusin 1		
MFN2	Mitofusin 2		
